# Tissue and plasma expression of the angiogenic peptide adrenomedullin in breast cancer

**DOI:** 10.1038/sj.bjc.6601397

**Published:** 2003-11-11

**Authors:** M K Oehler, D C Fischer, M Orlowska-Volk, F Herrle, D G Kieback, M C P Rees, R Bicknell

**Affiliations:** 1 Molecular Angiogenesis Laboratory, Cancer Research UK, Weatherall Institute of Molecular Medicine, University of Oxford, Oxford OX3 9DS, UK; 2 Department of Obstetrics & Gynaecology, University of Freiburg, D-79106 Freiburg, Germany; 3 Department of Pathology, University of Freiburg, D-79106 Freiburg, Germany; 4 Nuffield Department of Obstetrics & Gynaecology, John Radcliffe Hospital, University of Oxford, Oxford OX3 9DU, UK

**Keywords:** adrenomedullin, angiogenesis, immunohistochemistry, radioimmunoassay, tumour marker

## Abstract

Adrenomedullin (ADM) is an angiogenic factor that has also been shown to be a mitogen and a hypoxia survival factor for tumour cells. These properties point to ADM as a potential promoter of human malignancies, but little data are available concerning the expression of ADM in human breast cancer. In the present work, we have examined ADM peptide expression in a series of malignant breast tumours by immunohistochemistry using a newly developed anti-ADM monoclonal antibody. In addition, ADM plasma concentrations in breast cancer patients and healthy controls were determined by radioimmunoassay. Of the examined breast cancer samples, 27/33 (82%) showed a moderate to strong staining intensity. ADM-peptide expression in breast tumours was significantly correlated with axillary lymph node metastasis (*P*=0.030). Analysis of ADM plasma concentrations showed no significant difference between the circulating ADM levels of breast cancer patients and healthy controls. However, a significant positive correlation was found between tumour size and plasma ADM levels (*r*=0.641, *P*=0.017). Moreover, ADM levels in breast cancer patients correlated with the presence of lymph node metastasis (*P*=0.002). In conclusion, we have shown for the first time that ADM peptide is widely expressed in breast cancer and that the degree of expression is associated with lymph node metastasis. ADM peptide in plasma of breast cancer patients reflects the size of the primary tumour, but is unlikely to be a useful tumour marker for the detection of breast cancer. Plasma ADM might represent an independent predictor of lymph node metastasis. The clinical implications of these findings remain to be evaluated.

Adrenomedullin (ADM) is a 52-amino-acid peptide belonging to the calcitonin gene peptide superfamily based on its slight homology with calcitonin gene-related peptide (CGRP) and amylin. It acts through the G protein-coupled receptor calcitonin receptor-like receptor (CRLR), with specificity for ADM being conferred by the receptor-associated modifying protein 2 (RAMP2) (Poyner *et al*, 2002).

Adrenomedullin has been implicated in the modulation of various physiological functions, ranging from vasorelaxation to acting as a regulator of cell growth (Hinson *et al*, 2000; Nikitenko *et al*, 2002). In 1998, we first showed ADM to be potently angiogenic using the chick chorioallantoic membrane assay (Zhao *et al*, 1998), and it has subsequently been shown to be protumorigenic by a number of groups employing both xenograft studies (Martinez *et al*, 2002; Oehler *et al*, 2002) and blocking antibodies (Ouafik *et al*, 2002). Adrenomedullin and cancer have recently been reviewed (Zudaire *et al*, 2003).

Adrenomedullin is a hypoxically induced peptide under control of the hypoxia-inducible transcription factor-1 (HIF-1), as are several other angiogenic factors such as vascular endothelial growth factor (VEGF) (Garayoa *et al*, 2000). We have previously shown that ADM acts as an antiapoptotic factor, antagonising hypoxic cell death of tumour cells (Oehler *et al*, 2001). The protumorigenic activity of ADM is also in accordance with recent data that have revealed a clinical significance of ADM overexpression in malignant human tumours. Thus, increased expression of ADM mRNA was associated with poor overall survival in ovarian cancer (Hata *et al*, 2000) and high Gleason's scores in human prostate cancer (Rocchi *et al*, 2001).

Circulating levels of ADM have been shown to be elevated in various disease states including solid tumours such as gastrointestinal malignancies (Ehlenz *et al*, 1997). As the ADM peptide is rapidly secreted once produced in cells (Kitamura *et al*, 2002), it is conceivable that ADM peptide in the blood stream of tumour patients reflects the ADM secretion from the malignancies. An involvement of ADM in breast cancer has to date not been clearly documented, however. No data are available concerning ADM peptide expression in breast malignancies or of circulating ADM peptide in breast cancer patients.

In this study, we have examined ADM peptide expression in breast cancers by means of a newly characterised monoclonal antibody. In addition, the plasma concentrations of ADM in patients with breast cancer have been determined. We then analysed if there were associations between ADM expression in breast cancer tissue or blood and the clinico-pathological properties of patients. We finally assessed if ADM could have potential as a tumour marker in breast cancer.

## MATERIALS AND METHODS

### Materials

Human ADM used as an immunogen was chemically synthesised by the Cancer Research UK Peptide Synthesis Laboratory, Lincoln's Inn Fields, London, UK. The specificity of the resulting monoclonal antibodies was confirmed against both this material and commercial human ADM (Bachem (UK) Ltd., St Helens, UK). The amylin and CGRP used in this study were also purchased from Bachem (UK) Ltd.

### Patients and healthy controls

#### Tissue samples

Breast carcinoma tissues were derived from 33 randomly selected patients who underwent surgery for primary breast cancer at the Department of Obstetrics and Gynaecology, University of Freiburg, Germany between the years 1993 and 1999. Therapy consisted of either a wide local excision or modified radical mastectomy followed by axillary lymph node dissection. In all, 12 patients had distant metastasis. The clinicopathological properties of the patients are presented in Table 2.

#### Plasma samples

*Breast cancer patients* Preoperative plasma samples of 20 patients who underwent surgery for primary breast cancer at the John Radcliffe Hospital, Oxford, UK between December 1999 and January 2000 were included in the study. Patients underwent either modified radical mastectomy or a wide local excision. All patients had an axillary lymph node dissection. None of the patients had distant metastases. The clinicopathological properties of the series are presented in Table 4. All human tissue was collected with full approval of the local Ethics Committee (COREC number COO.097 – Analysis of expression of proteins induced by hypoxia in primary breast cancer) and patient consent.

*Controls* Plasma samples from 18 healthy female volunteer blood donors attending the blood bank at the John Radcliffe Hospital, Oxford, UK were included in the study.

### Preparation of the anti-ADM monoclonal antibody 171

Female Balb/c mice were immunised subcutaneously with 100 *μ*g of ADM peptide on day 1 followed by three further immunisations of 50 *μ*g at fortnightly intervals. The fusion of the antibody-producing B cells from the immunised murine spleen with an NS-1 myeloma partner was carried out using a standard method (Harlow and Lane, 1988). Supernatants from the hybridoma culture were screened by antibody capture on multiwell plates coated with ADM. Bound antibodies were detected using a peroxidase-conjugated goat anti-mouse secondary antibody (Dako, Glostrup, Denmark). Hybridomas were subsequently screened by Western blotting against ADM, using peptide from conditioned medium from previously described Ishikawa cells stably transfected with the cDNA for the ADM-cDNA (Oehler *et al*, 2001).

### Elisa

A measure of 10 *μ*g of protein in 100 *μ*l of PBS was added to individual wells of a 96-well Nunc-Immuno™ plate (Nalge-Nunc, Denmark) having a MaxiSorp™ surface overnight at room temperature. Plates were then blocked for 1 h in 20% ‘Marvel’ in PBS at room temperature. After washing, the wells were incubated with appropriate dilutions of hybridoma supernatant in PBS for 1 h at room temperature, washed and then incubated for a further hour at room temperature with goat anti-mouse horseradish peroxidase (DAKO, Denmark) diluted 1/2500 in PBS. Plates were washed again, blocked with 10% ‘Marvel’ in PBS for 10 min, washed and then treated with 100 *μ*l of 3,3′,5,5′-tetramethylbenzidine (TMB) ligand substrate system for ELISA (Sigma, Poole, UK) for periods of up to 30 min. Reactions were quenched with 50 *μ*l of 0.5 M sulphuric acid and the absorbance was read at 450 nm.

### Western blot analysis

For immunoblotting, proteins were resolved by SDS–PAGE in a 10% gel under reducing conditions and transferred onto Immobilon PVDF membranes (Immobilon PVDF – Millipore, Watford, UK) by semidry electroblotting. The PVDF membrane was blocked in PBS containing 0.1% Tween-20 and 5% nonfat milk before applying the monoclonal antibody 171 in a 1 : 500 dilution for 1 h. After washing in TBS containing 0.1% Tween, binding was visualised by a horseradish peroxidase-conjugated goat anti-mouse antibody (Dako, Glostrup, Denmark) followed by chemiluminescence detection with ECL Western blotting reagents (Amersham, Little Chalfont, UK).

### Adrenomedullin-transfected RL95.2 cells

Stable transfection of the endometrial carcinoma cell line RL95.2 with the cDNA for ADM followed by the selection of the ADM overexpressing cell clones RL-ADM1 and RL-ADM2 has been previously published (Oehler *et al*, 2002).

For immunostaining, the transfected cells were fixed in 10% neutral-buffered formalin, pelleted and embedded in paraffin. Sections (4 *μ*m) were cut onto silane-coated slides. These were stained according to the following protocol for the immunohistochemical analysis of tumour samples.

### Immunohistochemistry

Immunohistochemical analysis of tumour samples was performed according to standard techniques. Briefly, paraffin sections were deparaffinised in xylene and hydrated through a series of graded alcohols. Endogenous peroxidase was blocked with 0.3% hydrogen peroxide and nonspecific binding prevented by incubation with 10% normal horse serum diluted with phosphate-buffered saline (PBS). The slides were then incubated with the anti-ADM antibody 171 in a titre 1 : 100 diluted with PBS containing 1% normal horse serum for 2 h at 37°C. A commercially available avidin–biotin immunoperoxidase kit (Vectastain Elite anti-mouse ABC kit, Vector Laboratories, Burlingame, CA, USA) was then used to visualise bound antibody. The colour reaction was produced with DAB in the presence of 0.03% hydrogen peroxide. Counterstaining was carried out with haematoxylin. Normal kidney tissue was employed as a positive control, as it is known to express ADM-peptide consitutively (Asada *et al*, 1999).

Immunohistochemical staining was evaluated by two independent observers using an arbitrary staining score. Staining intensity was determined on the scale: 0=none, I=weak, II=moderate, III=strong.

### Radioimmunoassay

Blood samples were collected into chilled tubes with disodium-EDTA and aprotinin. Plasma was obtained by immediate centrifugation. The samples were stored at −80°C until further processing.

Plasma ADM concentrations were measured with a commercial competitive radioimmunoassay (RIA) (Peninsula Laboratories, Merseyside, UK) following the manufacturer's instructions. Briefly, plasma samples were acidified with 1% trifluoroacetic acid (TFA) and centrifuged. The supernatants were loaded onto Sep-Pak C18 cartridges (Millipore Corp., Waters Chromatography, Milford, USA) that had been equilibrated with 60% acetonitrile in 1% TFA and 1% TFA. The cartridges were then washed with 1% TFA and peptides were eluted with 60% acetonitrile in 1% TFA. The eluates were lyophilised and resuspended in radioimmunoassay (RIA) buffer. Assay samples or standard were incubated with rabbit anti-human ADM 1-52 antiserum overnight at 4°C. This antiserum is highly specific for human ADM and does not cross-react with rat ADM, human CGRP, amylin, endothelin-1, alpha atrial natriuretic peptide (1–28) or brain natriuretic peptide-32. ^125^I-ADM (15 000 counts per minute (c.p.m.)) was added to each tube and incubated for another 24 h. Antibody-bound ADM was precipitated using goat anti-rabbit antiserum. After centrifugation, the c.p.m. in the supernatant was measured with the gamma counter. The concentration of ADM was determined from a standard curve, which was constructed using serial dilutions of synthetic ADM. The detection limit was 1 pg tube^−1^. All plasma samples were processed as duplicates in a single assay to avoid interassay variability. The intra-assay coefficient of variation was 8%.

### Statistical analysis

Statistics were performed using the SPSS for Windows software package (SPSS Inc., Chicago, USA). The Pearson product–moment correlation was applied for analysis of associations between variables, and the Mann–Whitney U test and Kruskall–Wallis ANOVA, respectively, were employed for comparison of groups. *P*<0.05 was considered to be statistically significant.

## RESULTS

### Characterisation of the anti-ADM monoclonal antibody 171

**Figure 1:**
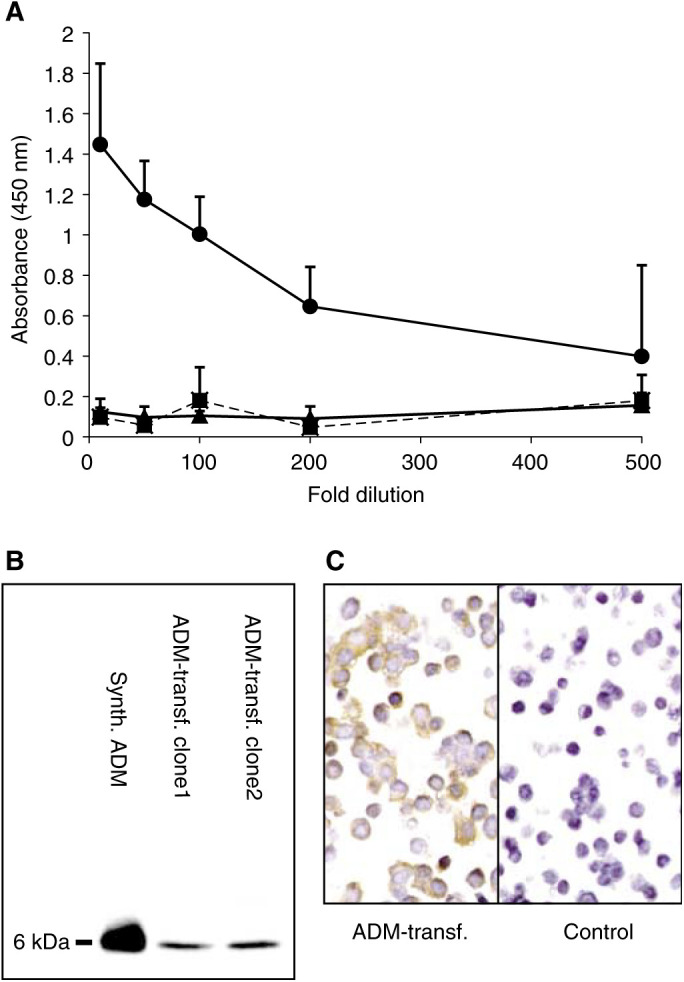
Characterisation of the anti-ADM monoclonal antibody 171. (**A**) Solid-phase ELISA using antibody 171 against ADM (circle), amylin (square) and CGRP (triangle). (**B**) Immunoblotting of supernatant from transfected ADM-overexpressing RL95.2 cell clones and synthetic ADM. (**C**) Immunostaining of ADM-overexpressing RL95.2 cell clones and controls. (Brown indicates immunostaining.)

Monoclonal antibodies from the fusion were screened by solid-state ELISA. Antibody 171 reacted strongly with ADM, but failed to recognise the related amylin or CGRP peptides (Figure 1A

). Antibody 171 was further found to detect synthetic ADM and the peptide in the conditioned media of ADM-transfected RL95.2 cells by Western blotting (Figure 1B). ADM-transfected RL95.2 cells showed a strong immunohistochemical labelling with this antibody while RL95.2 cells transfected with the empty vector were unstained (Figure 1C). Immunohistochemistry of normal kidney tissue with the antibody 171 stained distal tubules, collecting tubules and glomerular capillaries as previously described with anti-ADM polyclonal antisera.

### Adrenomedullin peptide expression in breast carcinomas

Of the examined breast cancer samples, 27/33 (82%) showed moderate to strong staining intensity. Of the remaining samples, only two tumours were negative for ADM (Table 1

**Table 1:** ADM peptide expression in breast carcinomas (IHC score)

	**ADM expression (IHC score)**			
**Total cases**	**0**	**I**	**II**	**III**
*n*=33	2 (6%)	4 (12%)	11 (33%)	16 (48%)

). In the breast malignancies, ADM staining was homogeneous and detectable mainly in the cytoplasm of the neoplastic cells (Figure 2

**Figure 2:**
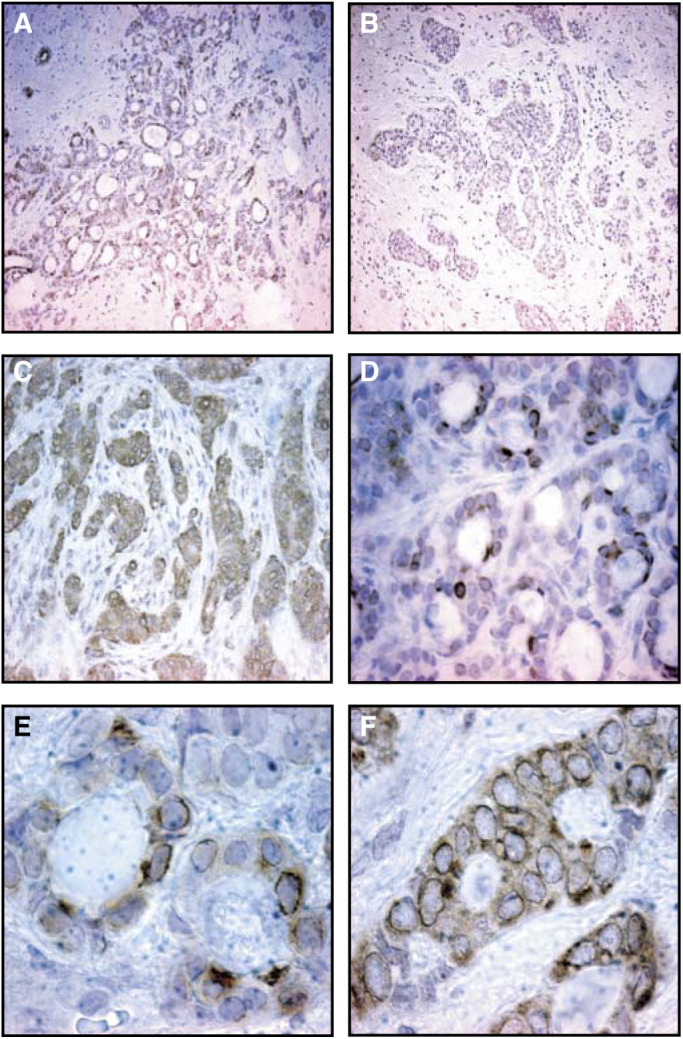
Immunostaining of breast carcinomas with the anti-ADM monoclonal antibody 171. (**A**) Moderate staining (× 100), (**B**) weak staining (× 100), (**C**) strong staining (× 200), (**D**) moderate staining (× 400), (**E**) moderate staining (× 1000), (**F**) strong staining (× 1000). (Brown indicates immunostaining.)

).

### ADM tissue expression and clinicopathologic features of breast cancer

#### Patients

**Figure 3:**
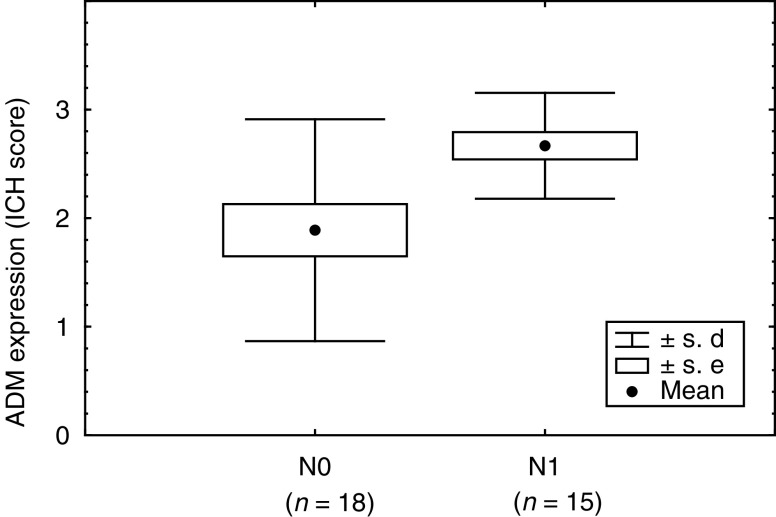
ADM expression in breast tumours of patients with (N1) or without (N0) lymph node metastasis.

Patients with axillary lymph node metastasis were found to have tumours with significantly higher ADM-peptide expression then patients with no lymphatic metastasis (N1: 2.70 (±0.49) (mean, s.d.) *vs* N0: 1.89 (±1.02) pg ml^−1^ (mean, s.d.), *P*=0.030, Mann–Whitney *U* test) (Figure 3

). ADM expression in breast cancers did not correlate with menopausal status, extent of tumour, distant metastasis, grading, ER or PR expression (Table 2

**Table 2:** Clinicopathological features and adrenomedullin (ADM) peptide expression (IHC score) in breast cancer tissue samples

**Clinicopathological features^a^**	**ADM expression (IHC score) mean (stdev)**	***P*-value**
Menopausal status		
premenopausal (*n*=10)	2.10 (±0.99)	NS^b^
postmenopausal (*n*=23)	2.30 (±0.88)	
Extent of tumour		
pT1 (*n*=14)	2.14 (±1.09)	NS
pT2 (*n*=11)	2.18 (±0.87)	
pT3 (*n*=2)	2.00 (±0.00)	
pT4 (*n*=6)	2.67 (±0.52)	

Nodal status		
pN0 (*n*=18)	1.89 (±1.02)	0.030
pN1 (*n*=15)	2.70 (±0.49)	
Metastasis		
M0 (*n*=21)	2.05 (±1.02)	NS
M1 (*n*=12)	2.58 (±0.51)	
Grading		
G1 (*n*=3)	2.33 (±0.58)	NS
G2 (*n*=9)	2.33 (±0.70)	
G3 (*n*=18)	2.11 (±1.80)	
ER		
Neg (*n*=5)	2.20 (±0.84)	NS
Pos (*n*=25)	2.24 (±0.97)	
PR		
Neg (*n*=8)	2.38 (±0.74)	NS
Pos (*n*=22)	2.18 (±1.00)	

a Where total numbers for each variable do not equal 33, data were unavailable.

b NS=non significant.

).

### Plasma ADM levels in breast cancer patients and controls

There was no statistically significant difference between ADM plasma concentrations of breast cancer patients 132.2±74.3 pg ml^−1^ (mean, s.d.) *vs* controls 107.9±32.7 pg ml^−1^, *P*>0.05; Kruskall–Wallis ANOVA) (Table 3

**Table 3:** Adrenomedullin peptide concentration in plasma of normal controls and breast cancer patients

**Group**	**Plasma ADM (pg/ml) mean ±s.d. (range)**	***P*-value**
Normal controls (*n*=18)	107.9±32.7 (59–192)	
Breast cancer patients (*n*=20)	132.2±74.3 (28–313)	NS^a^
pT1 (*n*=12)	90.9±50.1 (28–210)	NS
pT2 (*n*=7)	182.9±69.9 (128–313)	0.004^b^
pT3 (*n*=1)	209.0±0.0	

a NS=nonsignificant, *P*>0.05.

b Normal controls *vs* T2+T3.

). However, when the patients were stratified for tumour size, patients with larger breast tumours (≥T2) were found to express significantly higher ADM concentrations than healthy controls (≥T2: 186.2±65.4 pg ml^−1^ (mean, s.d.) *vs* controls: 107.9±32.7 pg ml^−1^ (mean, s.d.); *P*=0.004, Mann–Whitney *U* test) (Figure 4

**Figure 4:**
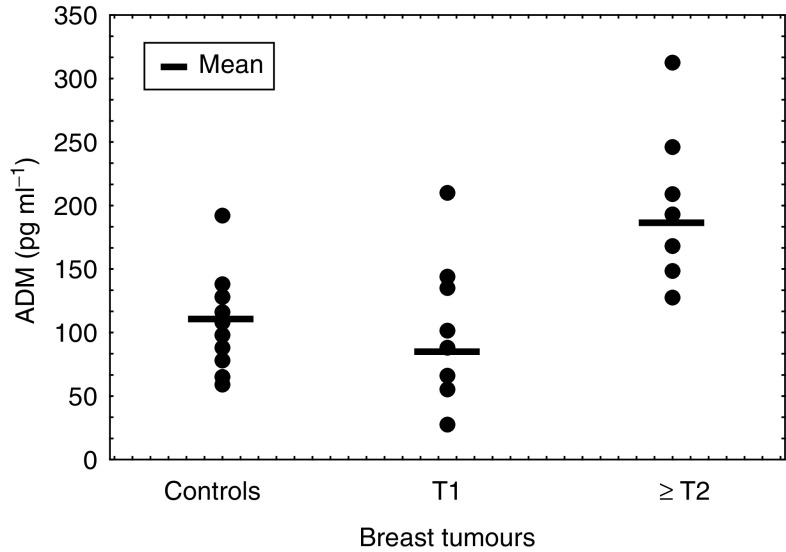
Plasma ADM concentrations of healthy controls *vs* breast cancer patients with T1 or ⩾ T2 tumours.

).

### Plasma ADM levels and clinico-pathologic features of breast cancer patients

**Figure 5:**
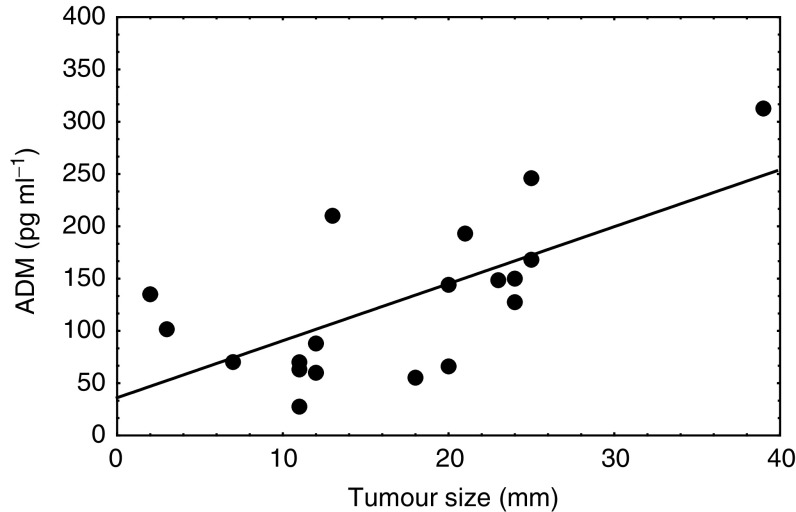
Correlation between tumour size and plasma ADM in breast cancer patients.

When analysing plasma ADM levels and clinicopathologic features of breast cancer patients, a significant positive correlation between tumour size and plasma ADM levels was observed (*r*=0.641, *P*=0.017, Pearson's product–moment correlation) (Figure 5

). Consequently, patients with larger tumours had significantly higher ADM plasma levels than patients with small volume disease (pT1: 90.9±50.1 pg ml^−1^ (mean, s.d.) *vs* pT2: 182.9±69.9 pg ml^−1^ *vs* pT3: 209.0±0.0 pg ml^−1^, *P*=0.019, Kruskall–Wallis ANOVA) (Table 4

**Table 4:** Clinicopathological features and ADM peptide concentration in plasma of breast cancer patients (*n*=20)

**Clinicopathological features**	**Plasma ADM (pg ml^−1^) mean ±s.d. (range)**	***P*-value**
Menopausal status		
premenopausal (*n*=3)	167.6±131.7 (55–313)	NS^a^
postmenopausal (*n*=17)	126.0±64.2 (28–246)	

Tumour size		
pT1 (*n*=12)	90.9±50.1 (28–210)	0.019
pT2 (*n*=7)	182.9±69.9 (128–313)	
pT3 (*n*=1)	209.0±0.0	

Nodal status		
pN0 (*n*=10)	81.4±35.9 (28–149)	0.002
pN1 (*n*=10)	183.1±68.1 (63–313)	

Histological type		
Ductal (*n*=18)	126.0±75.6 (28–313)	NS
Lobular (*n*=2)	188.5±29.0 (168–210)	

Grading		
G1 (*n*=2)	173.0±53.0 (135–210)	NS
G2 (*n*=12)	105.9±65.4 (28–246)	
G3 (*n*=6)	171.7±82.7 (66–313)	

ER		
Neg (*n*=9)	155.8±105.7 (60–313)	NS
Pos (*n*=11)	120.3±69.0 (28–246)	

a NS=nonsignificant, *P*>0.05.

).

**Figure 6:**
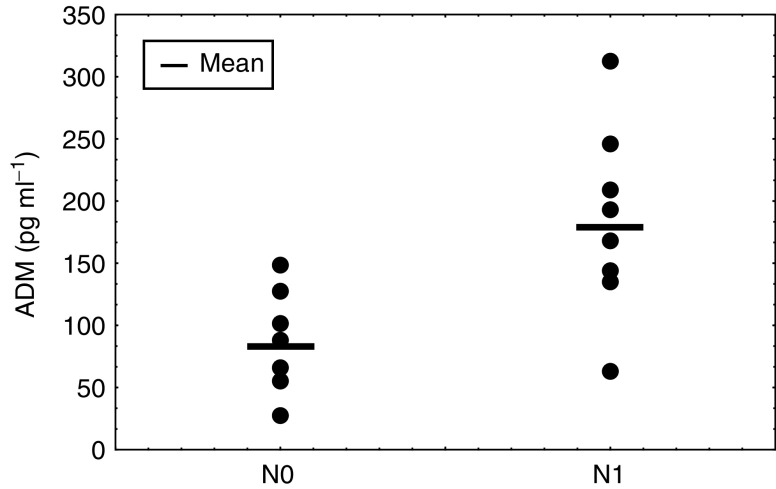
Plasma ADM concentrations in breast cancer patients with (N1) or without (N0) lymph node metastasis.

Furthermore, ADM levels in breast cancer patients correlated with the presence of lymph node metastasis (pN0: 81.4±35.9 pg ml^−1^ (mean, s.d.) *vs* pN1:183.1±68.1 (pg ml^−1^); *P*=0.002, Mann–Whitney *U* test) (Figure 6

).

The plasma concentrations of ADM in patients with breast cancer did not correlate with menopausal state, histological tumour type, grading or steroid receptor expression (Table 4).

## DISCUSSION

In this study, we have prepared an anti-ADM monoclonal antibody to assess the expression of ADM in malignant breast tissue. To our knowledge, no commercial monoclonal antibody against ADM is currently available and most of the previous immunohistochemical studies have employed polyclonal antisera (Washimine *et al*, 1995; Welsch *et al*, 2002). Our experiments with the antibody 171 have shown that it is suitable for Western blotting and immunostaining on formalin-fixed pathological specimens. Immunostaining in control tissue was comparable to results from previous studies using polyclonal antisera (Asada *et al*, 1999).

The series of breast tumours that we have examined shows that ADM is expressed in the majority of the carcinomas. The intensity of ADM expression, however, varied considerably in the malignancies. Staining of tumours was usually homogeneous and cytoplasmic in distribution.

This is the first study reporting ADM expression in breast cancers. Adrenomedullin immunostaining has previously been described for normal human and mouse mammary gland tissue (Jahnke *et al*, 1997; Welsch *et al*, 2002). In normal human breast tissue, both milk ducts as well as lobules were positive for ADM. As ADM immunopositivity was identified in basal parts of epithelial cells in glands and secretory granule-like structures were visualised by electron microscopy in these cells, it was suggested that ADM might be secreted into the blood stream acting as an endocrine signal (Jahnke *et al*, 1997).

In addition to peptide expression, ADM mRNA expression has also been previously examined in breast tissue. A recent study found ADM to be preferentially expressed in the terminal end buds (TEBs) of mammary glands of young rats (Chakravary *et al*, 2000). TEBs are composed of highly proliferative cells thought to be most susceptible to neoplastic transformation. It may therefore be hypothesised that although ADM is involved in normal mammary gland development, it may also have a role in breast cancer when aberrantly expressed.

Analysis of ADM expression and the clinicopathologic features of breast cancer patients showed that axillary lymph node metastasis significantly correlated with the intensity of ADM-peptide expression in tumours. These findings suggest a potential role of ADM in the development of lymph node metastasis in breast cancer.

Considering that ADM can function as an angiogenic factor and an apoptosis-survival factor, there are different mechanisms by which ADM might lead to the increased metastatic potential of malignant cells in breast cancer.

Adrenomedullin has been reported to be a survival factor for malignant cells under hypoxia or serum deprivation by upregulation of the antiapoptotic factors like Bcl-2 and Stat3 or downregulation of proapoptotic factors like Bax and Bid (Oehler *et al*, 2001; Martinez *et al*, 2002). Clinical studies have shown that Bcl-2 expression with loss of apoptosis are important determinants of lymph node metastasis in breast cancer (Sierra *et al*, 1996). It is thought that loss of apoptosis allows accumulation of genetic alterations affecting tumour-suppressor genes or oncogenes, resulting in the selection of more aggressive cell clones with the capability of lymphatic spread (Sierra *et al*, 2000). It is assumed that lymphatics contain low concentrations of oxygen and do not provide an adequate matrix for proliferation (Hockel *et al*, 1999). Tumour cells overexpressing ADM and possessing antiapoptotic potential, however, are more likely to be able to proliferate in lymphatic vascular spaces and in the subcapsular sinus of lymph nodes as the first step of lymph node metastasis.

Angiogenesis has also been thought to promote lymphatic spread of breast cancer (Saaristo *et al*, 2000). Hypoxia due to rapid tumour growth and defective microcirculation is very common in breast cancer and is known to be a driving force behind angiogenesis (Vaupel *et al*, 1991). Of the many angiogenic polypeptides now identified it appears that those induced by hypoxia are key players in tumour angiogenesis. Adrenomedullin upregulation under hypoxia has been shown to be controlled by the hypoxia-inducible transcription factor-1 (HIF-1) (Semenza, 2000), which is overexpressed in a high percentage of primary breast cancers (Zhong *et al*, 1999).

Adrenomedullin overexpressing tumours are characterised by an increased vascularity (Oehler *et al*, 2002). Several studies have shown that the probability of metastasis is correlated with the vascular density of primary tumours (Weidner *et al*, 1991). In ADM overexpressing breast cancers, increased angiogenesis might enhance the opportunity of tumour cells to gain access to the lymphatic system and to metastasise. In addition, the angiogenic potential of ADM overexpressing cells might increase the probability of tumour cells trapped in the lymphatic capillaries to induce neovascularisation and to give rise to macroscopic tumour growth.

In the immunohistochemical analysis, we were unable to find a correlation between ADM expression and tumour grade. Other groups, however, found a significant association between histological grading and ADM when analysing ovarian malignancies (Hata *et al*, 2000). Whether this observation is tumour-type specific, or whether we could not detect this association due to the limited numbers of cases included in our study is unclear.

In the second part of our study, we have analysed the plasma ADM values of breast cancer patients and healthy controls. The mean plasma ADM concentration for healthy subjects was 107.9±32.7 pg ml^−1^ (mean±s.d.). This value is in accordance to concentrations described by other groups (Garcia-Unzueta *et al*, 1998; Cases and Esforzando, 2000). A meta-analysis comparing 17 studies measuring plasma ADM in healthy subjects obtained by different immunoassays, however, showed a wide range of ADM concentrations in healthy women (Hinson *et al*, 2000). This observation shows that ADM assays still have to be standardised and that the range of normal ADM plasma concentrations remains to be defined.

When we analysed plasma ADM levels and clinicopathologic features of breast cancer patients, a significant positive correlation between tumour size and plasma ADM levels was observed. These results suggest that the source of circulating plasma ADM in those patients were indeed the breast malignancies. Active tumour growth, hypoxia and the associated overexpression of ADM might be responsible for the increased production and release of the peptide. In addition to such properties, ADM might have an adaptive function for tumours by increasing the intratumoral blood flow through its well-known vasodilative properties (Hinson *et al*, 2000).

Comparing circulating ADM levels of breast cancer patients and controls, ADM levels of patients with small (T1) cancers were similar to those of healthy controls. As a result, ADM is unlikely to be a useful marker with which to screen for breast cancer.

Nevertheless, patients with large tumours (>2 cm) had significantly higher ADM plasma concentrations than healthy controls or patients with small tumours (2 cm). It seems that malignant breast lesions require a critical size until a significant increase of secreted ADM is measurable in blood.

Increased expression of circulating ADM in cancer patients has also previously been described for colon and lung malignancies (Ehlenz *et al*, 1997). Furthermore, patients with Cushing's disease resulting from pituitary adenomas were found to have significantly higher circulating ADM levels than healthy controls. After surgical removal of the adenomas, ADM levels decreased to normal levels, indicating that the tumours were the main source of circulating ADM in those patients (Letizia *et al*, 2000).

We also found axillary lymph node metastasis to be significantly correlated with increased ADM plasma levels. Although the incidence of axillary lymph node involvement is well known to increase as a function of tumour size (Barth *et al*, 1997), we did not find this association in our series (data not shown). ADM plasma levels might therefore be an independent predictor of axillary lymph node metastasis in breast cancer.

In conclusion, we have shown for the first time that ADM peptide is widely expressed in breast malignancies. Tissue expression is associated with lymph node metastasis, possibly reflecting a more aggressive tumour type. ADM peptide can be measured in the plasma of breast cancer patients and reflects the size of the primary tumour. According to our series, plasma ADM does not distinguish between early disease and healthy controls and is unlikely to be a useful tumour marker for the detection of breast cancer. Plasma ADM levels are elevated in patients with lymph node involvement and may represent an independent predictor of lymph node metastasis. Further clinical studies appear justified.
